# Patient-Reported Outcome Measures of a Novel Cortical Button System for Distal Biceps Tendon Repair: A Retrospective Study

**DOI:** 10.7759/cureus.38621

**Published:** 2023-05-06

**Authors:** Kate Shean, Alex Chowdhury, Katharine Wilcocks, Daniel Blyth, Ahmed Elmorsy

**Affiliations:** 1 Trauma and Orthopaedics, Salisbury NHS Foundation Trust, Salisbury, GBR

**Keywords:** endobutton, suture button, distal biceps tendon repair, distal biceps rupture, bicep tendon

## Abstract

Background

There are a number of different techniques available for the repair of distal biceps tendon ruptures. Recent evidence has revealed satisfactory clinical outcomes for suture button techniques.

Aims

The aim of this study was to determine if the ToggleLoc^TM^ soft tissue fixation device (Zimmer Biomet, Warsaw, Indiana) confers satisfactory clinical outcomes in the surgical management of distal biceps ruptures.

Methods

Twelve consecutive patients underwent distal biceps repair using the ToggleLoc^TM^ soft tissue fixation device over a two-year period. Patient-Reported Outcome Measures (PROMs) were collected by means of validated questionnaires on two occasions. Symptoms and function were quantified using the Disabilities of the Arm, Shoulder and Hand (DASH) score and the Oxford Elbow Score (OES). Patient-reported health scores were determined using the EQ-5D-3L (European Quality of Life 5 Dimensions 3 Level Version) questionnaire.

Results

The mean initial follow-up time was 10.4 months and the mean final follow-up time was 34.6 months. The mean DASH score at the initial follow-up was 5.9 (se = 3.6), compared to 2.9 (se = 1.0) at the final follow-up (p = 0.30). The mean OES at the initial follow-up was 91.5 (se = 4.1); and 91.5 (se = 5.2) at the final follow-up (p = 0.23). The mean EQ-5D-3L level sum score at the initial follow-up was 5.3 (se = 0.3); and 5.8 (se = 0.5) at the final follow-up (p = 0.34).

Discussion

The ToggleLoc^TM^ soft tissue fixation device confers satisfactory clinical outcomes, as determined by PROMS, in the surgical management of distal biceps ruptures.

## Introduction

Ruptures of the distal biceps tendon comprise a spectrum, from degenerative fissuring to complete tears (with or without detachment of the lacertus fibrosus). The typical acute mechanism is the traumatic eccentric loading of a flexed elbow [1]. Predisposing activities include weight lifting, wrestling, or a labour-intensive job [1]. Risk factors include age, smoking, obesity, use of corticosteroids and overuse; all of which may contribute to tendon degeneration [1,2]. An annual incidence of 1.2-5.4 per 100,000 is reported, with a predilection for men (96%) in their middle age (mean 46.3 years) [3,4]. The incidence is rising, with greater sporting participation and an increasingly active elderly demographic speculated to be the cause [5].

Patients with low physical demands and multiple comorbidities are more suited to conservative management; however, outcomes of this may include a decrease in the strength and endurance of supination and flexion [1]. Operative management is usually indicated in younger patients wishing for optimal function, faster recovery and return to sports [1]. The frequency of operative management is increasing. This is partly due to reports of a supination strength reduction of 37-40% and flexion strength reduction of 7-30% with conservative management [6,7]. Indeed, in a recent survey of shoulder surgeons in the United Kingdom, 83% of respondents reported repairing more than half of these injuries [8]. Systematic reviews and meta-analyses comparing non-operative versus operative management have suggested operative management to result in improved strength and better patient-reported outcomes [9,10].

There are various techniques utilised for the surgical management of distal biceps tendon ruptures [11,12]. These include bone tunnels, intraosseous screws, suture anchors and cortical buttons. A recent retrospective study of a single-incision suture button repair for chronic distal biceps ruptures found the technique to be effective in improving the range of movement and functional outcomes [13].

Aim

The aim of this study was to determine if the ToggleLoc^TM ^soft tissue fixation device confers satisfactory clinical outcomes in the surgical management of distal biceps ruptures.

## Materials and methods

Patients

Twelve consecutive patients who underwent surgical repair of their distal biceps, under the care of the senior author at a district general hospital between May 2017 and July 2019, were included in this retrospective study. Patients were identified as suitable for surgery by the surgeon. To further review the mechanism of injury, a clinical assessment and subsequent ultrasound scan were performed to confirm injury/tendon retraction. Patient outcomes were collated in January 2019 and March 2021.

Patients were contacted by telephone to complete patient-reported outcome measures. The team was unable to gain objective measurements, such as range of movement or strength, due to the restrictions on hospital attendance secondary to COVID-19 pandemic restrictions.

Zimmer Biomet ToggleLoc^TM^

The ToggleLoc^TM^ device is indicated for soft tissue to bone fixation for a number of injuries, including biceps tendon repair. It consists of a 2.9 mm titanium button, to be placed through a bicortical hole. Attached to the button is a zip suture, with an adjustable length. It does not require interference screw fixation and is thus suitable for a single incision approach.

Surgical technique

**Figure 1:**
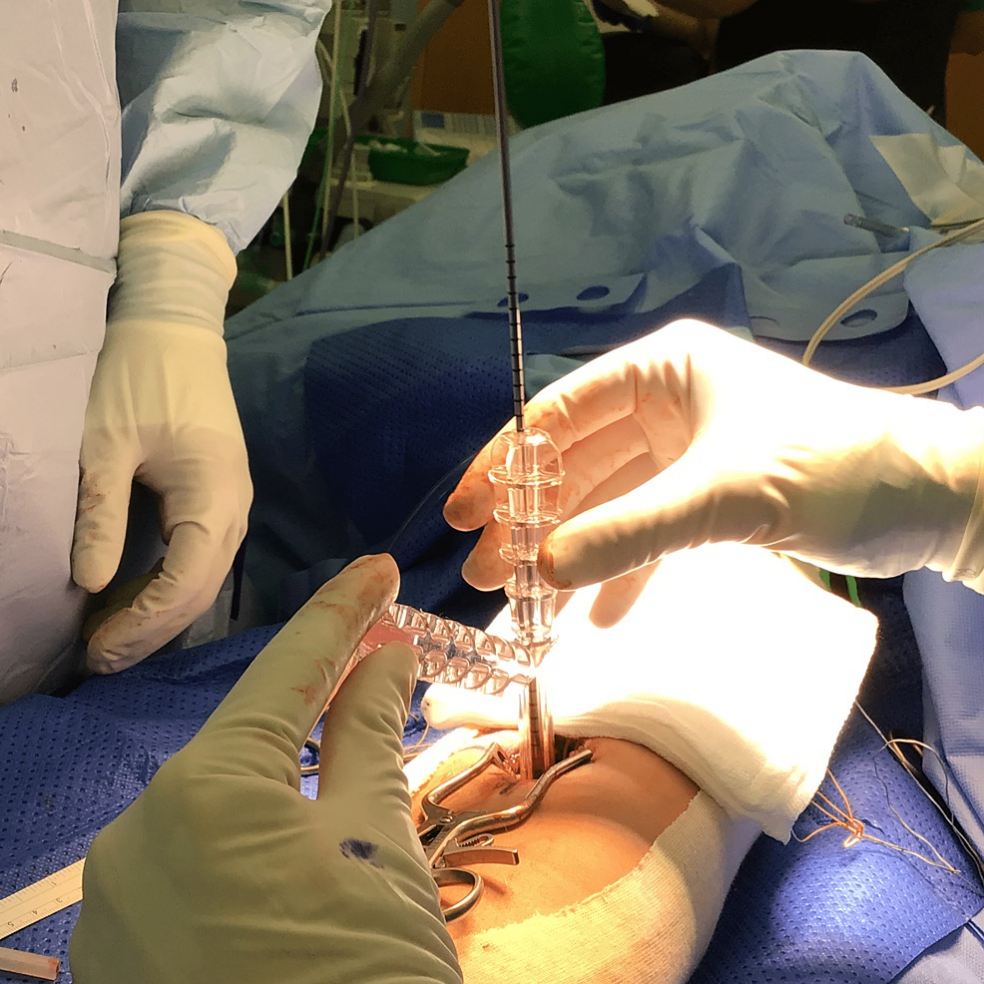
The insertion of the guide pin over the ToggleLoc guide

The patient is supine, with the arm abducted on an arm board. The arm is supinated to protect the posterior interosseous nerve (PIN) and to expose the radial tuberosity. Guided by an image intensifier, a longitudinal skin incision is centred on the radial tuberosity. The lateral antebrachial cutaneous nerve is identified and protected. The tendon sheath is opened to find the torn tendon end. In case of a tendon reaction, blunt dissection is used to identify the retracted end. In case of significant retraction, a separate incision on the anteromedial aspect of the distal part of the upper arm may be used to find the retracted tendon. Any maceration of the tendon end is debrided. The bicipital tuberosity is exposed. The ToggleLoc^TM^ guide is placed over the bicipital tuberosity. A 2.9 mm guide pin is drilled bicortically, directed in a slightly distal and ulnar direction to reduce the risk of a PIN injury (Figure 1).

**Figure 2:**
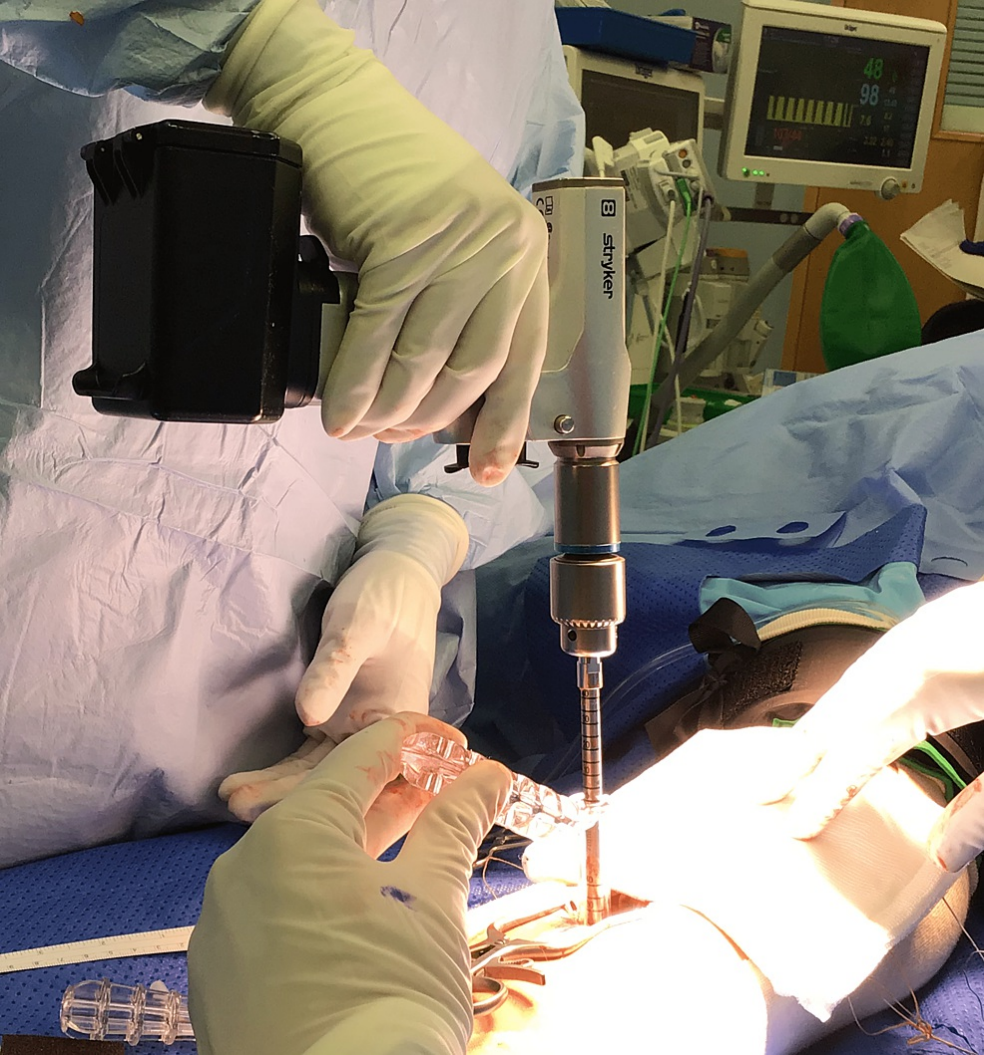
Unicortical reaming performed over the guide pin

The inner obturator is removed with the outer guide held in place. Unicortical reaming is performed over the guide pin (Figure 2). The guide pin is then removed, after which the Toggle Loc^TM^ implant is inserted through the slotted reamer. Once the implant passes through both cortices, the blue sutures are pulled to ensure that the endobutton rests on the posterior aspect of the radius. The reamer is then removed.

**Figure 3:**
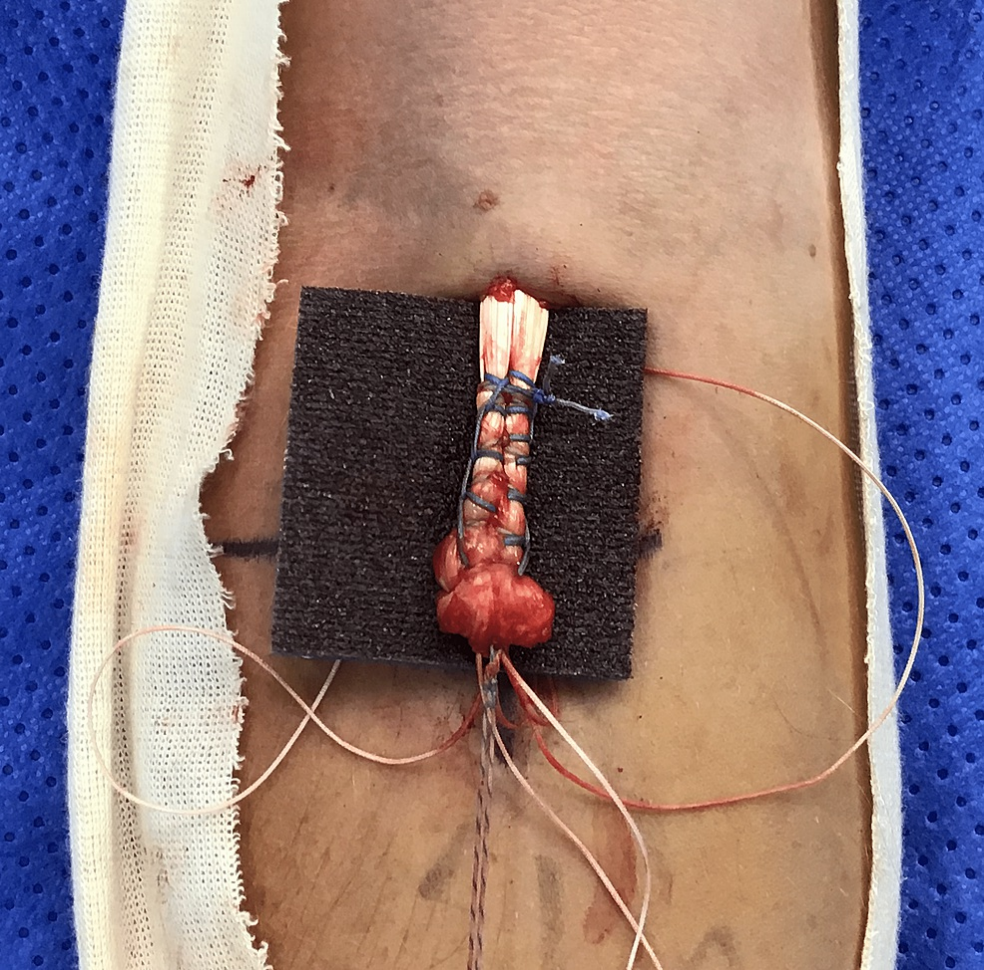
Krackow suture of the tendon, performed with the blue suture strands of the implant

**Figure 4:**
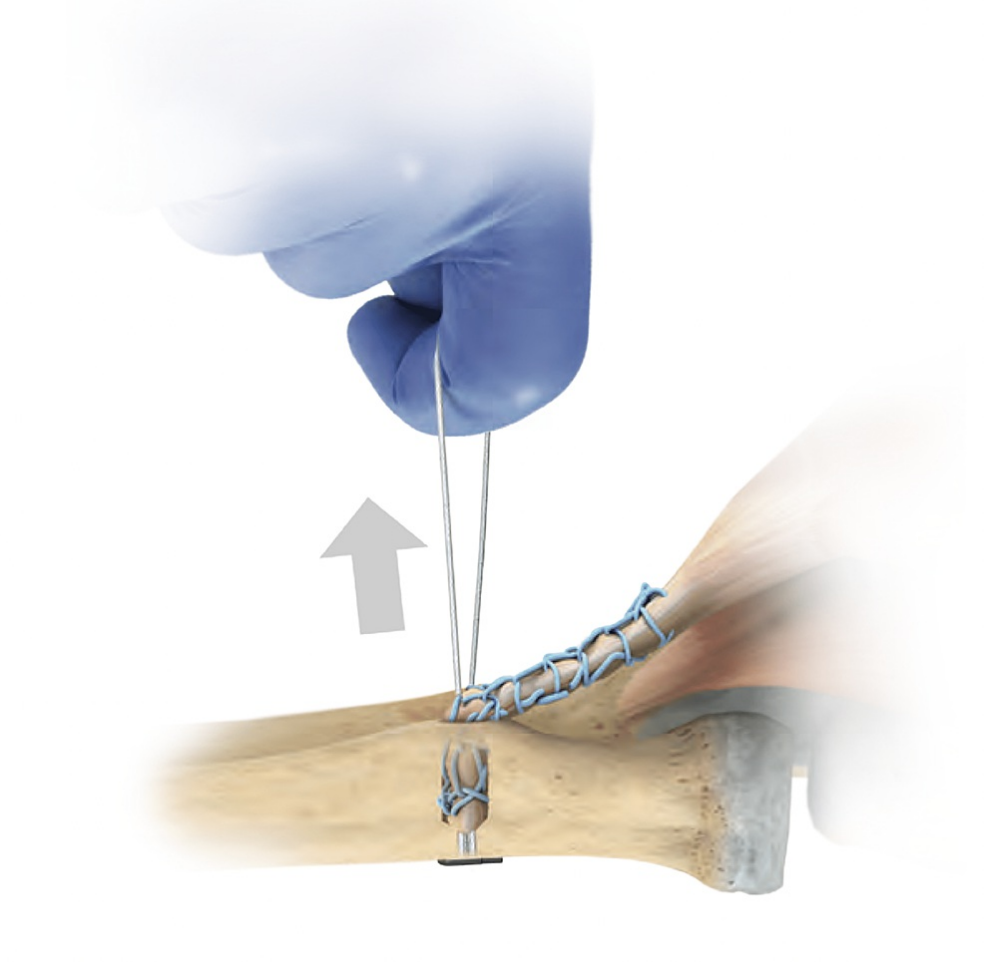
The white zipping strands are pulled to adequately tension the tendon Image used with permission from Zimmer Biomet

**Figure 5:**
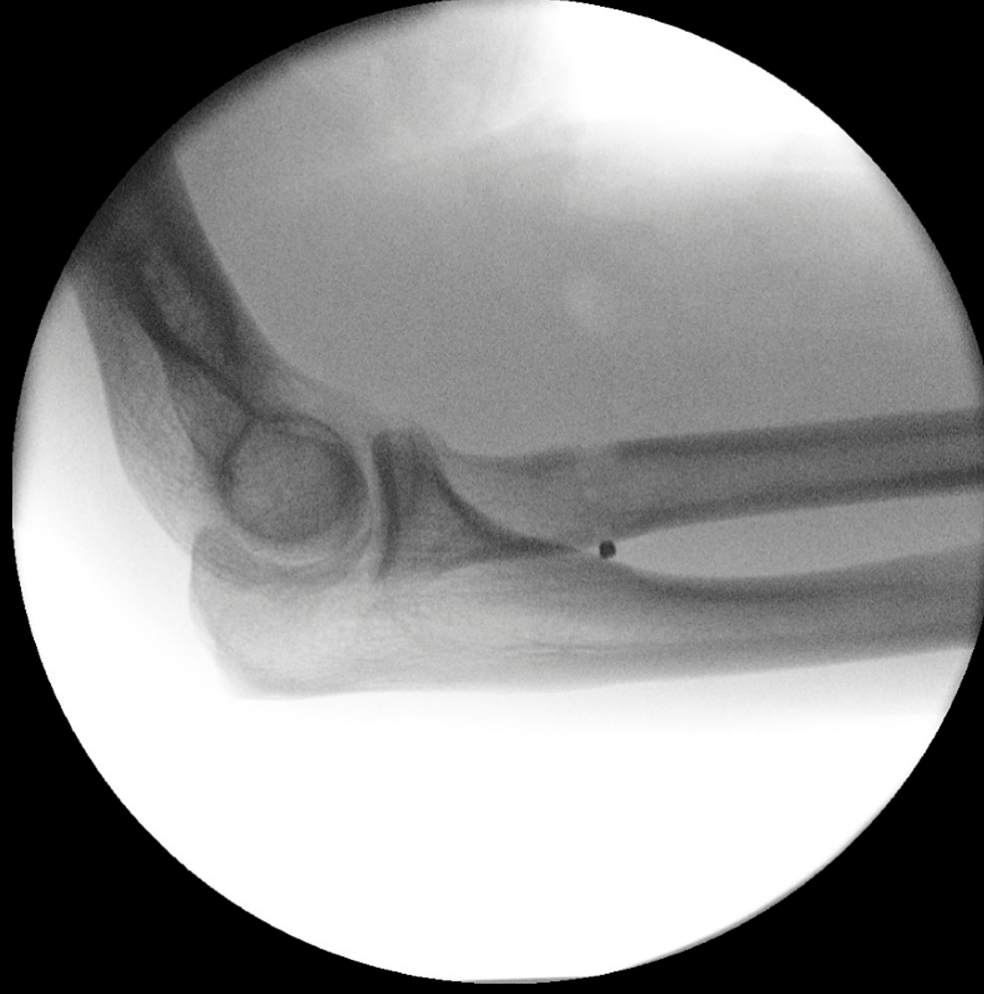
Image intensifier used to confirm the appropriate position of the button

The ExpressBraid^TM^ suture (Zimmer Biomet) is used to whipstitch the tendon from proximal to distal. After the removal of the needle, the two ExpressBraid^TM^ limbs are tied together around the blue connection strand. The tendon is attached to the implant using the two blue suture strands using a further Krackow technique, from distal to proximal (Figure 3). The tendon is then advanced down into the socket by pulling on the white zipping strands. When adequate tension has been achieved (Figure 4), the zipping strands are cut flush. Satisfactory positioning of the button, flush on the posterior radial cortex, is checked using the image intensifier (Figure 5).

Rehabilitation protocol

A trust-based rehabilitation program, developed using expert opinion and available literature, was followed for this cohort of patients under the supervision of experienced musculoskeletal physiotherapists (Table 1) [14].

**Table 1:** Rehabilitation protocol

Timescale	Rehabilitation
0-2 weeks	Patients are allowed to commence active-assisted elbow flexion, supination and pronation as tolerated. Active-assisted elbow extension limited to 30 degrees. Sling for comfort day & night.
2-6 weeks	Active elbow movements are allowed as tolerated between 2-6 weeks, with active extension limited to 30 degrees. Patients aiming to wean from wearing the sling during the day as tolerated & at night.
6-12 weeks	Begin to progress active-assisted elbow extension beyond 30 degrees, then actively as able. Commence grip strengthening. Wean fully from the sling. Return to driving once safe and comfortable to do so. No heavy lifting, pushing, pulling or biceps strengthening before 12 weeks.
12 weeks – 6 months	Commence elbow strengthening, initially isometric then progress. All movements allowed within comfort levels. Return to non-contact sports, including racquet sports.
6 months +	Resume heavy lifting and contact sports as comfortable.

Follow-up

The routine postoperative management following distal biceps tendon repair comprises a wound check by the GP practice at two weeks, followed by a consultant review in the clinic at six and 12 weeks. Patients are discharged at this time if satisfactory.

Outcome measures

Patient-Reported Outcome Measures (PROMs) were collected by means of validated questionnaires. Symptoms and functions were quantified using the Disabilities of the Arm, Shoulder and Hand (DASH) score [15] and the Oxford Elbow Score (OES) [16]. Patient-reported health scores were determined using the EQ-5D-3L (European Quality of Life 5 Dimensions 3 Level Version) questionnaire [17].

Statistics

Data were processed and means and standard errors were calculated using Microsoft Excel (Microsoft Office, Version 1908). To compare PROMs between the first and second follow-up times, the Wilcoxon signed-rank test was used, using SPSS (IBM SPSS Statistics for Windows, version 22.0. Armonk, NY).

## Results

Patient demographics

Twelve patients underwent a distal biceps tendon repair between May 2017 and July 2019. All 12 were successfully contacted for the collection of PROMs in January 2019, with a mean follow-up period of 10.4 months (SE 1.32). Each patient was again successfully contacted for a second follow-up, with a mean follow-up period of 34.6 months (SE = 1.37).

All participants were male. The mean age was 44 years old (range 27-57). Eleven participants were right-hand dominant. The dominant arm was injured in seven out of 12 (58%) participants. The mechanisms of injury are displayed in Table 2.

**Table 2:** Mechanisms of Injury

Activity	Number of participants
Rugby	5
Lifting heavy object	5
Avoiding assault	1
Fell off drag lift	1

Ultrasound scans were recorded for all participants contacted during this study. The distance of tendon retraction was not detailed for two of the sample. The mean retraction distance was 50.8 mm (range 18-80 mm).

DASH

The mean DASH score at the initial follow-up was 5.9 (SE = 3.6), compared to 2.9 (SE = 1.0) at the final follow-up (p = 0.30) (Table 3). Change in DASH score between follow-ups was recorded for all 12 participants. A minimal clinically important difference (MCID) value for DASH (≥ 10.81) was recorded for two participants between the first and second follow-up, with both participants reporting an improvement in symptoms/function [18]. Change in work module score between follow-ups was recorded for 10 participants. An MCID value was recorded for two of these participants: one showed clinically important improvement and one showed deterioration. Change in sports module score between follow-ups was recorded for seven participants. An MCID value was recorded for one of these participants, which suggested deterioration.

**Table 3:** PROMs at both follow-up timepoints * = Minimal Clinically Important Difference; DASH = Disabilities of the Arm, Shoulder and Hand; OES = Oxford Elbow Score; VAS = Visual Analog Scale; SE = Standard Error; PROMs = Patient-Reported Outcome Measures; EQ-5D-5L: European Quality of Life 5 Dimensions 3 Level Version

Patient no.	DASH (1^st^ follow-up)			DASH (2^nd^ follow-up)			OES (1^st^follow-up)	OES (2^nd^ follow-up)				EQ-5D-5L (1^st^ follow-up)		EQ-5D-5L (2^nd^ follow-up)	
	DASH (/100)	Optional Work (/100)	Optional Sport/Music (/100)	DASH (/100)	Optional Work (/100)	Optional Sport/Music (/100)	OES (/100)	OES (/100)	Function (/100)	Psycho/social /100)	Pain /100)	Descriptive score (5-25)	VAS health status (/100)	Descriptive score (5-25)	VAS health status (/100)
1	19.2	62.5	75	2.59*	75*	n/a	64.58	45.8	62.5	12.5	62.5	5	70	10	75
2	2.5	6.25	6.25	3.33	0	n/a	93.75	100	100	100	100	5	78	5	100
3	41.38	100	12.5	12.06*	0*	25*	60.42	62.5	62.5	56.25	68.75	8	97	9	80
4	0	0	0	0.83	0	0	100	100	100	100	100	5	60	5	70
5	4.17	0	n/a	0.83	0	n/a	97.92	100	100	100	100	5	85	5	95
6	0	0	0	0	0	0	100	100	100	100	100	5	75	5	90
7	1.67	0	0	1.67	0	0	100	98	100	100	94	5	100	5	100
8	0	0	0	0	0	0	100	100	100	100	100	5	75	5	90
9	1.67	0	25	0.83	n/a	n/a	95.83	100	100	100	100	6	75	5	75
10	0	0	0	0	0	0	100	100	100	100	100	5	70	5	80
11	0	0	0	0	0	0	97.92	100	100	100	100	5	68	5	79
12	0	n/a	n/a	1.67	0	0	87.5	100	100	100	100	5	90	6	60
mean (SE)	5.9 (3.6)	15.3 (10.2)	11.9 (7.5)	2.9 (1.0)	7.5 (6.8)	2.8 (2.8)	91.5 (4.1)	91.5 (5.2)	93.75 (4.2)	89.06 (7.9)	74.5 (3.8)	5.3 (0.3)	78.6 (3.5)	5.8 (0.5)	82.8 (3.6)

OES

The mean OES at the initial follow-up was 91.5 (SE = 4.1) and 91.5 (SE = 5.2) on the second follow-up (p = 0.23) (Table 3). Considering individual participants, the OES score improved between the first and second follow-ups for 50% of participants. Thirty-three per cent (33%) of participants neither improved nor deteriorated; 17% reported deterioration in symptoms. The MCID value of 10 was not realized for any of the participants between the first and second follow-ups.

EQ-5D-5L

The mean EQ-5D-3L level sum score at the initial follow-up was 5.3 (SE = 0.3) and 5.8 (SE = 0.5) at the second follow-up (p = 0.34) (Table 3). The mean EQ-Visual Analog Health Score at the initial follow-up was 78.6 (SE = 3.5) and 82.8 (SE = 3.6) at the second follow-up (p = 0.36). The scores indicate a good perceived health status for all participants.

Complications

One patient had a postoperative wound infection, necessitating a washout and debridement a month later. One patient reported mild forearm numbness, which was self-limiting. Another patient experienced the loss of FDP function in their little finger. This was extensively investigated with USS, magnetic resonance imaging and electrophysiology, though no cause was found.

## Discussion

All of the participants in this study reported satisfactory clinical outcomes, as determined by PROMs, following the surgical repair of distal biceps ruptures using the ToggleLoc^TM^ soft tissue fixation device. These outcomes were maintained between the initial and second follow-ups. Satisfactory outcomes were noticeable as early as two months postoperatively. The success of surgery does not appear to be influenced by the distance of retraction. Complications following surgery were reported for three participants. These did not appear to detrimentally affect the patient-reported outcome following surgery. Objective strength measurements were not recorded for this cohort due to COVID-19 restrictions on clinic follow-ups.

Debate exists between a single and double incision approach for distal biceps repair. The two-incision approach (usually using bone tunnels or suture anchors) has been suggested to yield superior supination strength and endurance, through easier anatomical restoration [12]. It has also been suggested to result in a reduced risk of nerve injury. However, heterotopic ossification (HO) has been noted as a major complication [12]. The single incision approach has been suggested to be associated with less HO, though an increased risk of nerve injury. The results of this study, using a single anterior approach, support this, with one incidence of self-limiting forearm numbness and no cases of HO.

There is no consensus on the optimal treatment for total or partial distal biceps tendon repairs [19]. A review of evidence of the different fixation methods suggests stronger initial fixation strength of the cortical button and the cortical button/interference screw construction, compared to suture anchor and interference screw alone [20]. Initial fixation strength allows early active mobilisation and loading, possibly improving outcomes [20]. The lack of anatomical reinsertion options when using a single-incision technique prompted the development of new alternative fixation methods, including a double intramedullary button fixation device [12]. A study of cortical button, transosseous suture and suture anchor techniques concluded all of these methods to yield good functional results [19].

Endobutton techniques have been found to give good results in the repair of complete and partial distal biceps tears, with regard to strength, range of movement and functional outcomes [21]. A previous study of the ToggleLoc^TM^ device demonstrated good clinical and functional results, with a relatively rare incidence of posterior interosseous nerve palsy [22]. The findings in this study were comparable.

This study is subject to a number of limitations. There was a reasonably small cohort of patients (n=12). There was no inclusion of estimated baseline function pre-injury to compare to. It was not feasible to gain real-time measurement of the participants’ baseline function, due to the traumatic nature of this injury. A consideration for future studies could be to include participants’ subjective estimation of their baseline. Due to restrictions imposed on face-to-face clinic appointments during the COVID-19 pandemic, we were unable to bring participants to the clinic to obtain objective strength or range of movement measures. This study, therefore, only uses subjective data from patient-reported outcome measures.

## Conclusions

This study was performed to evaluate the outcomes of a novel endobutton device in the management of distal biceps tendon ruptures. The ToggleLoc^TM ^soft tissue fixation device, a cortical button system, is inserted through a single incision technique. Detailed here are the technique, the rehabilitation protocol and the clinical outcome measures in a series of 12 patients. The ToggleLoc^TM^ soft tissue fixation device confers satisfactory clinical outcomes, as determined by patient-reported outcome measures, in the surgical management of distal biceps ruptures.

## References

[1] Caekebeke P, Duerinckx J, van Riet R (2021). Acute complete and partial distal biceps tendon ruptures: what have we learned? A review. EFORT Open Rev.

[2] Vishwanathan K, Soni K (2021). Distal biceps rupture: evaluation and management. J Clin Orthop Trauma.

[3] Kelly MP, Perkinson SG, Ablove RH, Tueting JL (2015). Distal biceps tendon ruptures: an epidemiological analysis using a large population database. Am J Sports Med.

[4] Safran MR, Graham SM (2002). Distal biceps tendon ruptures: incidence, demographics, and the effect of smoking. Clin Orthop Relat Res.

[5] Launonen AP, Huttunen TT, Lepola V (2020). Distal biceps tendon rupture surgery: changing incidence in Finnish and Swedish men between 1997 and 2016. J Hand Surg Am.

[6] Morrey BF, Askew LJ, An KN (1985). Rupture of the distal tendon of the biceps brachii. A biomechanical study. J Bone Joint Surg Am.

[7] Freeman CR, McCormick KR, Mahoney D, Baratz M, Lubahn JD (2009). Nonoperative treatment of distal biceps tendon ruptures compared with a historical control group. J Bone Joint Surg Am.

[8] Baldwin MJ, Watts AC, Peach CA, Phadnis J, Singh H, Gwilym SE (2022). Treatment of acute distal biceps tendon ruptures - a survey of the British Elbow and Shoulder Society surgical membership. Shoulder Elbow.

[9] Looney AM, Day J, Bodendorfer BM, Wang D, Fryar CM, Murphy JP, Chang ES (2022). Operative vs. nonoperative treatment of distal biceps ruptures: a systematic review and meta-analysis. J Shoulder Elbow Surg.

[10] Cuzzolin M, Secco D, Guerra E, Altamura SA, Filardo G, Candrian C (2021). Operative versus nonoperative management for distal biceps brachii tendon lesions: a systematic review and meta-analysis. Orthop J Sports Med.

[11] Watson JN, Moretti VM, Schwindel L, Hutchinson MR (2014). Repair techniques for acute distal biceps tendon ruptures: a systematic review. J Bone Joint Surg Am.

[12] Caekebeke P, Bain G, van Riet RP (2020). Distal biceps tendon repair with the endobutton technique. Surgical Techniques for Trauma and Sports Related Injuries of the Elbow.

[13] Zeman CA, Mueller JD, Sanderson BR, Gluck JS (2020). Chronic distal biceps avulsion treated with suture button. J Shoulder Elbow Surg.

[14] (2022). Salisbury NHS Foundation Trust. Physiotherapy rehabilitation guidelines for patients following distal biceps tendon reattachment. https://viewer.microguide.global/guide/1000000308.

[15] (2022). Institute for Work and Health. The DASH outcome measure. https://dash.iwh.on.ca/about-dash.

[16] (2022). Oxford University Innovation: The Oxford Elbow Score (OES). https://innovation.ox.ac.uk/outcome-measures/the-oxford-elbow-score-oes/.

[17] (2022). EuroQol Research Foundation: EQ-5d-3L. https://euroqol.org/eq-5d-instruments/eq-5d-3l-about/.

[18] Franchignoni F, Vercelli S, Giordano A, Sartorio F, Bravini E, Ferriero G (2014). Minimal clinically important difference of the disabilities of the arm, shoulder and hand outcome measure (DASH) and its shortened version (QuickDASH). J Orthop Sports Phys Ther.

[19] Lang NW, Bukaty A, Sturz GD, Platzer P, Joestl J (2018). Treatment of primary total distal biceps tendon rupture using cortical button, transosseus fixation and suture anchor: a single center experience. Orthop Traumatol Surg Res.

[20] Tjoumakaris FP, Bradley JP (2020). Distal biceps injuries. Clin Sports Med.

[21] Siebenlist S, Schmitt A, Imhoff AB (2019). Intramedullary cortical button repair for distal biceps tendon rupture: a single-center experience. J Hand Surg Am.

[22] Alech-Tournier F, Elkholti K, Locquet V (2019). Outcomes of distal biceps tendon reattachment using the ToggleLoc™ fixation device with ZipLoop™ technology with single mini-open technique. Eur J Orthop Surg Traumatol.

